# Investigation of the Association between Air Pollution and Non-Alcoholic Fatty Liver Disease in the European Population: A Mendelian Randomization Study

**DOI:** 10.3390/toxics12030228

**Published:** 2024-03-21

**Authors:** Jing Yang, Yaqi Zhang, Yin Yuan, Zhongyang Xie, Lanjuan Li

**Affiliations:** 1 State Key Laboratory for Diagnosis and Treatment of Infectious Diseases, National Clinical Research Center for Infectious Diseases, National Medical Center for Infectious Diseases, Collaborative Innovation Center for Diagnosis and Treatment of Infectious Diseases, The First Affiliated Hospital, Zhejiang University School of Medicine, 79 Qingchun Rd., Hangzhou 310003, China; 11818049@zju.edu.cn (J.Y.); 12118240@zju.edu.cn (Y.Z.); yuanyin@zju.edu.cn (Y.Y.); zyxie@zju.edu.cn (Z.X.); 2 Department of Biology, The David H. Koch Institute for Integrative Cancer Research at MIT, Massachusetts Institute of Technology, Cambridge, MA 02139, USA

**Keywords:** air pollution, NAFLD, UK Biobank, Finngen, Mendelian randomization

## Abstract

Non-alcoholic fatty liver disease (NAFLD) is currently the most prevalent chronic liver disease worldwide. At the same time, the relationship between air pollution and the likelihood of developing NAFLD has been a subject of debate due to conflicting findings in previous observational research. Our objective was to examine the potential correlation between air pollutant levels and the risk of NAFLD in the European population by employing a two-sample Mendelian randomization (MR) analysis. The UK Biobank Consortium provided the summary statistics for various air pollution indicators (PM_2.5_, PM_2.5_ absorbance, PM_2.5–10_, PM_10_, NO_2_, and NO_x_). Additionally, information on NAFLD was obtained from three studies, including one derivation set and two validation sets. Heterogeneity, pleiotropy, and sensitivity analyses were performed under different MR frameworks, and instrumental variables associated with confounders (such as education, smoking, alcohol, and BMI) were detected by tools. In the derivation set, causal relationships between PM_2.5_, NO_2_, and NAFLD were observed in univariable Mendelian randomization (UVMR) (Odds Ratio (OR) = 1.99, 95% confidence interval (95% CI) = [1.22–3.22], *p* = 0.005; OR = 2.08, 95% CI = [1.27–3.40], *p* = 0.004, respectively). After adjustment for air pollutants or alcohol intake frequency in multivariable Mendelian randomization (MVMR), the above genetic correlations disappeared. In validation sets, the null associations remained in UVMR. Our findings from MR analysis using genetic data did not provide evidence for a causal association between air pollution and NAFLD in the European population. The associations observed in epidemiological studies could be partly attributed to confounders.

## 1. Introduction

The escalating prevalence of non-alcoholic fatty liver disease (NAFLD) has made it a major global health issue. The estimated worldwide incidence rate of NAFLD stands at approximately 24% [1]. Notably, dietary patterns and lifestyle choices have been recognized as crucial elements linked to the emergence of NAFLD, with countries exhibiting a higher consumption of high-calorie diets experiencing a greater incidence of the disease. Furthermore, NAFLD independently contributes to the likelihood of various comorbidities, including hypertension, type 2 diabetes (T2D), and cardiovascular diseases (CVDs) [2,3,4]. Although metabolic disorders have been established as the primary risk factor for NAFLD, increasing evidence supports the view that exposure to certain environmental factors may have a profound impact on liver disease, including NAFLD.

A matter of concern is the detrimental impact of air pollution on human health, as over 80% of city dwellers are exposed to air pollutants whose levels exceed the thresholds set by the World Health Organization (WHO) [5]. Air pollutants typically encompass particulate matter (PM) and specific chemical gases. PM can be categorized based on its physical diameter: particulate matter with aerodynamic diameters ≤ 2.5 μm (PM_2.5_); particulate matter with diameters ≤ 10 μm and >2.5 μm (PM_2.5–10_); particulate matter with diameters > 10 μm (PM_10_). The chemical substances include gases such as nitrogen dioxide (NO_2_), nitrogen oxides (NO_x_), sulfur dioxide (SO_2_), ozone (O_3_), and so on. PM_2.5_, originating from vehicle exhaust emissions, industrial emissions, and combustion processes, is the pollutant most commonly detected among them. Air pollutants have been officially classified by the International Agency for Research on Cancer (IARC) as the main category of substances that cause cancer in humans. A number of studies have consistently demonstrated correlations between air pollution and various forms of cancer, including lung cancer [6], gastrointestinal cancer [7], ovarian cancer [8], and others [9]. As a kind of well-established and extensively widespread industrial contaminant, PM_2.5_ possesses the capacity to deeply infiltrate the respiratory system, disturb the exchange of gases, and infiltrate the bloodstream, consequently presenting a substantial risk in relation to respiratory ailments, cardiovascular conditions, and mental well-being. In the year 2015, surrounding PM_2.5_ became the fifth leading cause of death, accounting for 4.2 million fatalities and 103.1 million disability-adjusted life-years (DALYs). The aforementioned statistics represented 7.6% of all deaths worldwide and 4.2% of global DALYs, with a noteworthy majority (59%) concentrated in the eastern and southern Asian regions [10].

It is crucial to acknowledge that the emergence of numerous illnesses is an intricate consequence arising from the interaction among various genetic factors, lifestyle choices, and environmental elements. Previous research has indicated that genetic polymorphism plays a vital part in investigating the effects of contaminants on different physiological and immune functions in the human body. For instance, a study rooted in genetics has demonstrated that women with the *GPX4*-rs376102 AC/CC genotype exhibit heightened vulnerability to atmospheric pollutants, consequently increasing the likelihood of preterm births [11]. Similarly, elderly individuals with *PARP4* G-C-G and *ERCC1* T-C are prone to increased levels of fasting blood sugar when exposed to PM_2.5_, PM_2.5–10_, and PM_10_ [12]. Moreover, the presence of indoor PM_2.5_ and environmental tobacco smoke during pregnancy greatly increases the occurrence of lower respiratory tract infections in newborns who possess the *GSTM1* null, *GSTP1*-rs1695 AG/GG, or *Nrf2*-rs6726395 GG genotypes [13].

While several observational studies have suggested a correlation between air pollution and NAFLD, it is crucial to obtain further evidence to establish a more robust causal relationship. This requirement stems from the potential influence of confounding factors, misclassification, and the inherent difficulties associated with reverse causality that are prevalent in observational study designs. MR research is usually likened to a natural randomized controlled trial (RCT), as it relies on the random assignment of genetic variations/alleles from parents during meiosis in pregnancy. Conceptually, MR research shares similarities with a RCT, wherein participants are randomly placed in different experimental groups. This implies that there is no discernible association between individuals possessing a specific genetic variation and exposure factors, thus rendering it a natural random allocation. Moreover, due to the inherent stability of the human genome once established, confounding biases that are arduous to regulate (e.g., lifestyle, economic status) are significantly diminished, thereby yielding more dependable causal evidence. At the pragmatic level of implementation, MR studies employ genetic variations (single nucleotide polymorphisms, SNPs) as instrumental variables to deduce a causal relationship between exposures and outcomes. Additionally, genome-wide association studies (GWASs) furnish an extensive repertoire of genetic variation analysis data related to human diseases by testing the correlation between millions of genetic variations and disease outcomes. To establish the causal connection between air pollution and NAFLD, a two-sample MR (TSMR) analysis was performed.

## 2. Materials and Methods

### 2.1. Study Design

**Figure 1:**
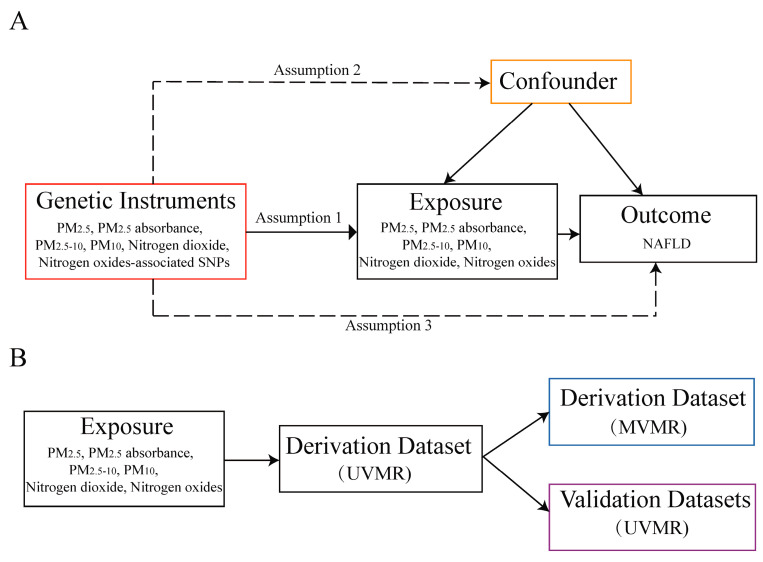
MR assumptions and the design flow chart of this study. (**A**) MR assumptions: assumptions 1, 2, and 3. The solid line represents direct putative causal effects that air pollution genetic instrumental variants are reliably associated with air pollutant levels and influence the risk of NAFLD through the exposures in assumption 1. The dotted line represents that genetic instrumental variants are not associated with any measured or unmeasured confounders and do not influence the risk of NAFLD through other pathways in assumptions 2 and 3, respectively. (**B**) The flow chart of the study design. MR, Mendelian randomization; PM, particulate matter; NAFLD, non-alcoholic fatty liver disease; UVMR, univariable Mendelian randomization; MVMR, multivariable Mendelian randomization.

The genetic variations employed in this analysis are required to adhere to the three assumptions of Mendelian randomization (MR) [14], as presented in Figure 1. Firstly, the genetic instrumental variables (IVs) related to air pollution, such as PM_2.5_, PM_2.5_ absorbance, PM_2.5–10_, PM_10_, NO_2_, and NO_x_ exposure levels, exhibit significant associations. Secondly, the relationship between these genetic IVs and NAFLD remains unaffected by confounding factors. Lastly, the genetic IVs solely influence the risk of NAFLD through exposure.

The study design depicted in Figure 1 provides an overview of the research methodology. The objective of this MR study was to examine the possible causal connection between the atmospheric contaminants (PM_2.5_, PM_2.5_ absorbance, PM_2.5–10_, PM_10_, NO_2_, and NO_x_) and NAFLD. Firstly, a derivation outcome set was obtained from the same source as the previous observational study [15], and a univariate Mendelian randomization (UVMR) study model was employed for discovery purposes. Subsequently, exposures with positive UVMR results and the confounding factors identified in the aforementioned observational study were included for multivariate Mendelian randomization (MVMR) analysis. Additionally, validation outcome sets from two distinct populations were employed to improve the overall dependability of this research. It is important to mention that this research followed the reporting recommendations specified in Strengthening observational studies using Mendelian randomization (STROBE-MR) [16]. The Supplementary Materials contain the STROBE-MR checklist for reference.

### 2.2. Data Sources

The summary datasets for the air pollution GWASs in Europe were obtained from the MRC-IEU, a unit of the Medical Research Council that has streamlined its process to perform GWASs on the imputed genetic dataset of the entire UK Biobank population of 500,000 individuals with efficiency, effectiveness, and uniformity. Land use regression (LUR) models were used to measure the relevant indicators for air pollution in Europe, which include PM_2.5_, PM_2.5_ absorbance, PM_2.5–10_, PM_10_, NO_2_, and NO_x_ [17]. Other datasets could also be found in the IEU open GWAS project, including the alcohol-intake frequency dataset. The NAFLD GWAS summary datasets were derived from three studies that exclusively included individuals of European descent (GWAS ID: ebi-a-GCST90054782 [18], ebi-a-GCST90091033 [19], finn-b-NAFLD [20]). Considering the distinct sources of the queues and different sample overlap ratios of these datasets, we employed the first dataset as the derivation dataset and the other two datasets as the validation datasets. Consisting of 10 centers in the United States, the eMERGE Network is a substantial genetic research establishment. With a population of around 200,000 Estonian adults, the Estonian Biobank is an organized biobank established by the Estonian Genome Center of the University of Tartu (EGCUT). FinnGen is an extensive research project that integrates inherent gene data obtained from recently gathered and existing samples from 400,000 participants in the Finnish biobank. It also incorporates digital health registers to offer innovative understandings of the genetics of human disorders [20].

The specific information of the datasets, such as cohort sources, diagnostic codes, and data adjustment methods, is listed in Table 1 and Table S1. All documents in this undertaking originated from the project website and were accessible to the general public. In the respective original studies, all participants provided informed consent. Therefore, there is no requirement for additional ethical authorization or a form of informed consent.

### 2.3. Selection of Instrumental Variables

To satisfy assumption 1, we implemented a selection procedure for the relevant SNPs for exposure, utilizing a widely recognized threshold of genome-wide significance (*p* < 5 × 10^−8^). However, only NO_2_ and NO_x_ exposures yielded an adequate number of SNPs. Previous research has suggested a limited potential for weak instrumental variable bias in MR analysis when employing linear regression of each genetic variant on risk variables at a screening threshold of *p* < 1 × 10^−5^ [21]. Based on the extant literature on MR studies related to PM_2.5_, we discovered that the threshold values for screening IVs were set at *p* < 1 × 10^−6^, *p* < 5 × 10^−6^, or even *p* < 1 × 10^−5^, which exceeded the usual range. As a result, we performed MR operations under two different threshold conditions (*p* < 1 × 10^−6^ and *p* < 1 × 10^−5^) to acquire a sufficient quantity of SNPs. The independence of SNPs was verified by implementing rigorous inclusion criteria (r^2^ ≤ 0.001; clumping window, 10,000 kb) without a proxy SNP in linkage disequilibrium. A harmonization procedure was undertaken to ascertain positive strand alleles and employ allele frequencies for palindromes as a means of quality control by R software.

To fulfill assumption 2, we used the PhenoScanner (http://www.phenoscanner.medschl.cam.ac.uk/) and GWASCatalog (https://www.ebi.ac.uk/gwas/) tools (accessed on 25 December 2023) to determine if there was a significant correlation between IVs and the risk factors associated with NAFLD, including smoking, alcohol, BMI, T2DM, blood pressure, education, and other factors that had been confirmed to have a causal relationship with NAFLD in MR analyses published so far. For detailed information, please refer to Table S3. When one SNP was mixed with multiple variables, only the variable with the lowest *p*-value or the strongest level of clinical evidence was listed in the table.

To meet assumption 3, we used the above tools and conducted MR Steiger filtering to monitor the direction of causation [22] and calculated the proportions of variance explained by exposures (R^2^) relying on prior investigations. In addition, the F-statistic (F = β^2^/SE^2^) for each SNP listed in Table S2 was calculated to present the strength of IVs, and a value greater than 10 was deemed satisfactory, indicating poor chances of weak instrumental bias [23].

### 2.4. Mendelian Randomization Analysis

The primary approach used for evaluating the causal relationship was the inverse variance weighted (IVW) method, which employed an odds ratio (OR) as the effect value. In MR analysis, IVW employs a single genetic IV to estimate the causal effect using the Wald ratio. After that, several evaluations are meta-analyzed using a fixed-effect model, ensuring a reliable estimation of causality without directed pleiotropy. This approach is commonly employed and referenced in studies [21,24]. To improve accuracy and stability, we enhanced our verification by incorporating the MR-Egger regression, weighted median, weighted mode, simple mode, and MR Robust Adjusted Profile Score (MR-RAPS). By utilizing MR-RAPS, it becomes possible to incorporate numerous weak instruments that fall below the typical GWAS threshold, thereby enhancing the dependability of Cochran’s Q-statistic in detecting heterogeneity caused by pleiotropy. This is especially beneficial in reducing the false positive (or type I error) rate. Online calculations were performed to estimate the bias and type I error rate of MR with sample overlap (https://sb452.shinyapps.io/overlap/, accessed on 31 December 2023). Post hoc power calculations [25] for IVW-MR estimates were produced using an online MR power calculation tool (https://sb452.shinyapps.io/power/, accessed on 31 December 2023). Moreover, to mitigate the influence of variables that may distort the results in UVMR, MVMR was utilized to assess the relationship among exposures, confounding factors, and outcomes.

### 2.5. Sensitivity Analysis

Sensitivity analyses encompassed three components and were executed using several methods. Initially, we assessed the heterogeneity by employing Cochran’s Q test for the IVW approach, and a *p*-value less than 0.05 indicated the presence of heterogeneity among the chosen IVs [21,26]. In addition, we assessed horizontal pleiotropy by employing MR-Egger regression and MR-pleiotropy residual sum and outlier (MR-PRESSO) [27] to ensure compliance with assumptions 2 or 3. The MR-Egger regression model enables the estimation of corrected pleiotropic effects in a causal manner, assessing the null causality assumption based on the InSIDE (instrument strength independent of direct effect) assumption. When the *p*-value of the MR-Egger intercept was less than 0.05, we deemed the impact of SNPs linked to exposure factors on outcomes to be untrustworthy. The MR-PRESSO algorithm allows for a systematic evaluation of the impact of pleiotropy and identifies exceptional SNPs, while also offering a causal estimation by eliminating associated outliers. Thirdly, we employed the leave-one-out permutation test [28] to test if our findings were impacted by a specific SNP in order to eliminate chance errors from the selection of IVs. If the results of the MR study were significantly altered by excluding a single SNP, it suggests that this particular SNP might have a direct association with the results, thereby violating assumption 3.

If heterogeneity was detected without pleiotropy, the weight median method or the multiplicative random-effects inverse variance weighting (mre-IVW) method was chosen for analysis. If there was identification of horizontal pleiotropy but no heterogeneity, the MR-Egger method was chosen. While the MR-Egger method is recognized for its resilience against pleiotropy, it is also influenced by diminished statistical accuracy and increased likelihood of Type I error in practical applications [29]. Therefore, if the IVW approach yielded a significant outcome without any detected pleiotropy or heterogeneity, while the outcomes of alternative methods were not significant but exhibited beta values in the same direction, it could be considered a favorable outcome. Additionally, to provide further clarification, scatter plots, forest plots, and funnel plots were generated.

### 2.6. Statistical Analysis

R software (version 4.3.0) was utilized for all analyses, employing the packages “TwoSampleMR” (version 0.5.7), “MRPRESSO,” and “MR.RAPS”. The level of statistical significance for evidence was established at *p* < 0.05. It is important to acknowledge that no correction methods for multiple testing were employed. After taking into account the possible constraints of implementing corrections, such as the Bonferroni correction, this choice was made, as it may severely limit the detection of causal relationships. Multiple test corrections are not always applicable, particularly in exploratory studies [30]. Due to the investigative character of our study, which sought to reveal fresh associations and impacts, the application of various test adjustment techniques was considered unsuitable for accomplishing our goals.

## 3. Results

### 3.1. UVMR Results in the Derivation Dataset

**Figure 2:**
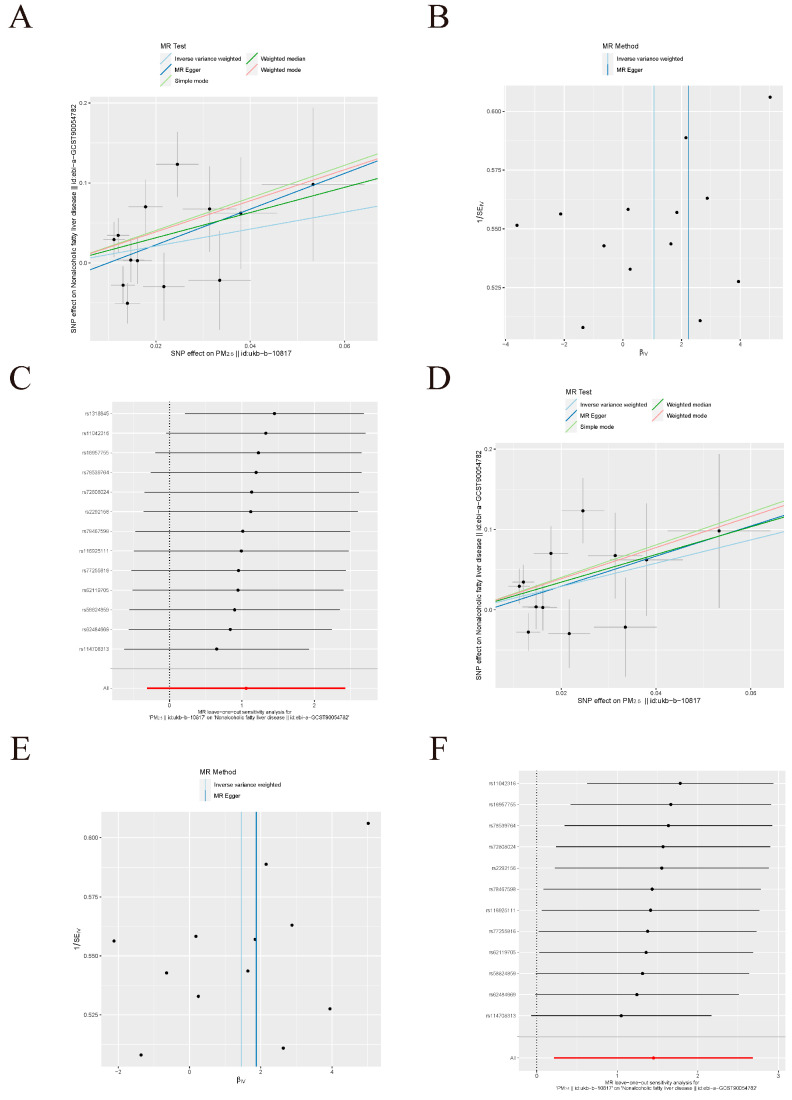
Scatter plots, funnel plots, and leaveone-out plots for causal SNP effects of PM_2.5_ on non-alcoholic fatty liver disease in the derivation dataset at level *p* < 1 × 10^−6^. (**A**) Scatter plot (**B**) Funnel plot (**C**) Leave-one-out plot for the exposure of PM_2.5_ before removing outlier-SNP. (**D**) Scatter plot (**E**) Funnel plot (**F**) Leave-one-out plot for the exposure of PM_2.5_ after removing outlier-SNP. The error bars indicate the 95% confidence interval (CI).

In the UVMR analysis of the derivation set, the screening threshold was set at *p* < 1×10^−6^ (as indicated in Table 2), and SNPs associated with confounding factors or NAFLD were excluded. The genetic prediction indicated that PM_2.5_ was linked to a higher risk of NAFLD (OR = 4.83, 95% CI = [1.03–22.65], Pweighted median = 0.046), with heterogeneity observed and no evidence of pleiotropy. Based on the leave-one-out plot illustrated in Figure 2, it was observed that rs1318845 stood out as an anomalous SNP. After removing it, the UVMR calculation was performed again. Currently, the IVW approach yielded a positive outcome (OR = 4.26, 95% CI = [1.24–14.64], P_IVW_ = 0.021), with the absence of heterogeneity or pleiotropy. Figure 2 displays the scatter plots, funnel plots, and leave-one-out plots.

**Figure 3:**
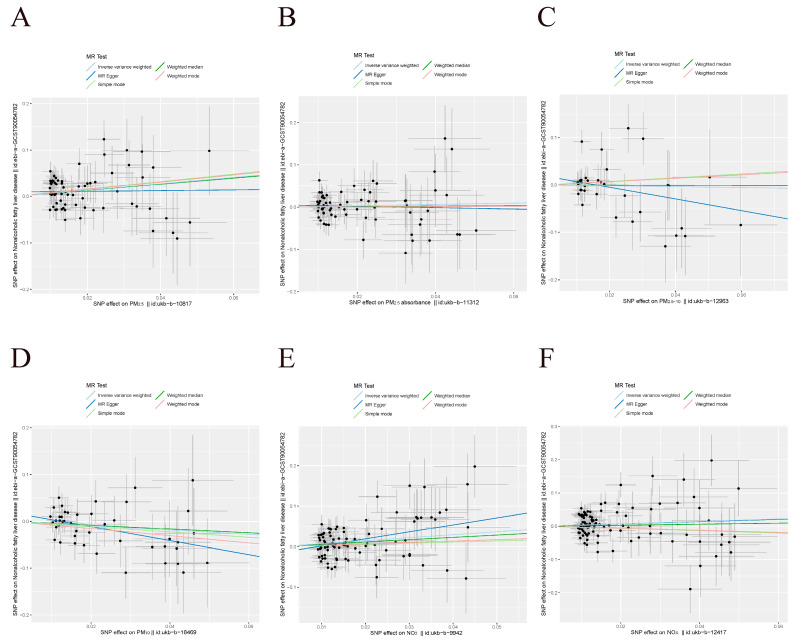
Scatter plots for visualizing the causal effects of air pollution on NAFLD in the derivation dataset at level *p* < 1 × 10^−5^. (**A**–**F**) Scatter plots for the exposure of PM_2.5_, PM_2.5_ absorbance, PM_2.5−10_, PM_10_, nitrogen dioxide, and nitrogen oxides, respectively. Each black point representing each SNP effect on the exposure (horizontal-axis) and outcome (vertical-axis) is plotted with error bars corresponding to standard error. The slope of each line corresponds to the combined estimate using different methods: inverse variance weighted (light blue line), MR-Egger (blue line), simple mode (light green line), weighted median (green line), and weighted mode (pink line).

In the analysis of the derivation set using the UVMR set at *p* < 1 × 10^−5^ (shown in Table 3 and Figure 3), it was observed that PM_2.5_ and NO_2_ had a connection with NAFLD (OR = 1.99, 95% CI = [1.22–3.22], P_IVW_ = 0.005; OR = 2.08, 95% CI = [1.27–3.40], P_IVW-mre_ = 0.004, respectively).

Moreover, there was no observed association between NAFLD and PM_2.5_ absorbance, which served as a substitute for carbonaceous elements in PM_2.5_. Table S4 showed that, when using the IVW, weighted median, MR-Egger, simple mode, weighted mode, and MR-RAPS methods, there was no indication of a causal connection between NAFLD and other air pollutants (IVW method, PM_2.5_ absorbance: *p* = 0.503; PM_2.5–10_: *p* = 0.813; PM_10_: *p* = 0.124; IVW-mre method, NO_x_: *p* = 0.159). Table S4 displays the outcomes of the prejudice and type I error rates in MR with sample overlap, along with post hoc power calculations. Steiger-MR found that the SNPs accounted for a greater amount of variability in exposure compared to the outcome.

### 3.2. MVMR Results in the Derivation Dataset

**Figure 4:**
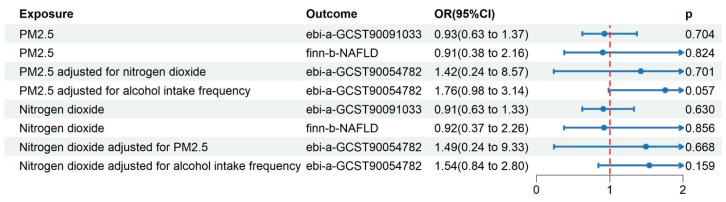
UVMR results of PM_2.5_ and Nitrogen dioxide on NAFLD in the validation datasets and MVMR results in the derivation dataset at level *p* < 1 × 10^−5^. UVMR, univariable Mendelian randomization; MVMR, multivariable Mendelian randomization.

To account for the influence caused by the interaction of PM_2.5_ and NO_2_, we conducted an MVMR analysis simultaneously considering PM_2.5_ and NO_2_ as exposures. As a result, the causal effects of PM_2.5_ and NO_2_ on NAFLD were absent after conducting MVMR analysis (OR = 1.42, 95% CI: 0.24–8.57, *p* = 0.701; OR = 1.49, 95% CI: 0.24–9.33, *p* = 0.668; respectively). At the same time, we accounted for the frequency of alcohol intake as a modifying factor for the relationship between air pollution and NAFLD risk. Subsequently, the MVMR analysis depicted in Figure 4 revealed that the connections between PM_2.5_ and NO_2_ with NAFLD (OR = 1.76, 95% CI = [0.98–3.14], *p* = 0.057; OR = 1.54, 95% CI = [0.84–2.80], *p* = 0.159, respectively) contradicted the estimates obtained from the UVMR analysis.

### 3.3. UVMR Results in the Validation Datasets

For further validation, we used two outcome datasets with different sample overlap ratios to perform UVMR. In the UVMR analysis of validation sets at *p* < 1 × 10^−5^, we found no causal relationship between PM_2.5_, NO_2_, and NAFLD using all MR methods (shown in Table S4). Steiger-MR found that the SNPs accounted for a greater amount of variability in exposure compared to the outcome.

## 4. Discussion

Epidemiological studies on air pollution and NAFLD have been conducted around the world. Research from Asia presented consistent results. In two separate studies, which included 23,170 and 90,086 Chinese individuals, it was discovered that different air pollutants were linked to advanced liver fibrosis (ALF) in patients with metabolic-associated fatty liver disease (MAFLD) and increased odds of MAFLD itself. Notably, PM_2.5_ emerged as the primary factor in these associations [31,32]. A study conducted in Taiwan involving around 35,000 Chinese Taiwanese individuals revealed that being exposed to PM_2.5_ was linked to a higher chance of developing NAFLD [33]. Additionally, a separate study with 351,852 participants discovered that prolonged exposure to PM_2.5_ might lead to higher levels of liver enzymes, particularly alanine aminotransferase (ALT) and γ-glutamyl transferase (γ-GT) [34]. Liver enzyme increases were also found to be linked to exposure to PM_10_ and CO in Korea [35]. Research from western countries presented contradictory results. According to research involving 2513 individuals from the Framingham (Massachusetts) Offspring Study and Third Generation Cohort, residing in proximity to main highways, instead of PM_2.5_, was found to have a probable connection with liver fat [36]. Conversely, a study conducted in Germany involving 4814 inhabitants indicated a positive correlation between prolonged exposure to air pollution and NAFLD. The study found that the most reliable connections were observed between PM_2.5_ and NAFLD. However, it did not establish a consistent link between air pollution exposure and an increased likelihood of advanced fibrosis [37]. Similarly, research involving 456,687 individuals residing in the United Kingdom discovered that the presence of PM_2.5_, PM_2.5–10_, PM_10_, NO_2_, and NO_x_ in the environment contributed to the additional hazard of NAFLD linked to air pollution scores. Furthermore, the impact of alcohol consumption acted as a modifying factor in the connection between these factors, as revealed by subgroup analysis. After adjusting for alcohol consumption and other covariates in the past 10 years, the majority of associations remained [15]. The derivation set of our study used the same sample source as the observational study and obtained similar results during the UVMR process. However, after adjusting for alcohol intake frequency as a confounding factor through MVMR, the previously discovered association disappeared. One possible reason was that the exposure sets and derivation outcome set used in this study were both from the UKB, so the results were affected by confounding factors during the UVMR process, which often occurred in single-sample Mendelian studies. Although we used parameter estimates to evaluate possible biases caused by sample overlap, and the results showed no unacceptable biases or inflated Type I error rates (Table S4), combining the MVMR results of the derivation set and the UVMR results of the validation sets, we still considered that the results in the derivation queue were false positive. In other words, the available data failed to substantiate the hypothesis of a causal association between air pollutants and NAFLD within the European population. Perhaps this was also the reason why PM_2.5_ absorbance, representing the composition of carbon elements, was not found to have a causal relationship with NAFLD in this study.

However, even so, we cannot completely deny the potential impact of air pollutants on NAFLD. One possible inference is that there exists a threshold effect, which has sparked discussion in the field of cardiovascular disease [38]. The PM_2.5_ level in the UKB datasets was 9.99 ± 1.06 μg/m^3^ (Table S1), with 50% falling below 10 μg/m^3^ and 90% below 12 μg/m^3^, which are limits established by the European Union and the United States Environmental Protection Agency, respectively. The baseline level of air pollutant concentrations might influence the analysis results, so regional differences may lead to different results.

The significant results in animal models may also be due to this reason. Researchers found obvious changes in liver morphology and function in mice after short-term exposure to large amounts of inhalable pollutants, with doses far exceeding those that humans could come into contact with in their daily lives. For a period of 30 days, Leah J. Schneider et al. subjected three-month-old male C57Bl/6 mice to either a low-fat or high-fat (HF) diet and exposed them to either filtered air (FA) or MVE (30 µg/m^3^ gasoline engine emissions + 70 µg/m^3^ diesel engine emissions) for 6 h per day. Histology findings showed that MVE exposure alone resulted in mild microvesicular steatosis and hepatocyte hypertrophy, compared to FA controls. Additionally, the mixed effect of HF diet and MVE exposure led to increased lipid accumulation, inflammatory infiltrates, and hepatocyte hypertrophy [39]. Hui-Hui Tan et al. exposed mice to PM_2.5_ at an average level of 85 µg/m^3^ for 6 weeks; it was discovered that exposure to PM significantly enhanced the secretion of IL-6 by isolated wild-type Kupffer cells. The increase in IL-6 secretion was up to seven times higher and showed a dependence on the dosage, whereas TLR4^−/−^ Kupffer cells did not exhibit the same response. The progression of NAFLD is significantly influenced by the activation of TLR in Kupffer cells, which are macrophages residing in the liver, leading to the production of pro-inflammatory cytokines. Dongxiao Ding et al. verified that a 3-day exposure to liposoluble extracts of PM_2.5_ at a concentration of 25 µg/cm^2^ caused the accumulation of lipids in HepG2 cells. This accumulation was linked to a reduction in the expression of miR-26a and a subsequent increase in the levels of fatty acid translocase (FAT, or CD36), whose increase resulted in enhanced uptake of free fatty acids (FFAs) [40].

At present, the disease progression of NAFLD from simple steatosis is elucidated by the “two-hit” theory. The “first hit” involves reversible and simple fat accumulation (fatty liver or steatosis). Excessive buildup of triglycerides (TG) in liver cells occurs due to heightened absorption of lipids and the synthesis of new lipids, inadequate breakdown of fatty acids oxidation (FAO), and diminished release of lipids [41]. The “second hit” encompasses various damages and conditions, such as inflammatory cytokines, oxidative stress, and toxins, promoting the advancement of NAFLD to non-alcoholic steatohepatitis (NASH), fibrosis, and hepatocellular carcinoma [42]. For example, augmented lipid content and impaired FAO facilitate the generation of reactive oxygen species and lipophilic lipid intermediates in hepatic cells, promoting oxidative stress and endoplasmic reticulum stress. Chronic oxidative stress initiates an inflammatory reaction, primarily through the activation of the JNK and NF-κB signaling pathways. This results in an increased production of pro-inflammatory proteins (i.e., IL-6 and TNF-α) transmitted by liver cells and non-parenchymal cells [43]. The continuous stimulation of pro-inflammatory reactions sustains a persistent state of inflammation, leading to the enlistment of additional immune cells and the initiation of cellular apoptosis and other mechanisms of cell demise. Cell damage and apoptosis can be promoted by non-triglyceride lipid substances, including long-chain fatty acids, and their products, such as ceramides and diacylglycerols, leading to the harmful effects of lipid accumulation in liver cells known as lipotoxicity [44]. Extended exposure to PM2.5 resulted in elevated insulin resistance, impaired glucose tolerance, peripheral inflammation, and dysarteriotony in mice induced by PM_2.5_. Moreover, the hepatic function and lipid accumulation in the liver were significantly influenced by the inflammation response and oxidative stress, as indicated by previous research [45]. This phenomenon can be described as a “second hit” for NAFLD. Other air pollutants have also been tested in animal models. Female mice exposed to NO_2_ experienced elevated levels of hepatic enzymes in their serum, resulting in liver dysfunction. However, this effect was not observed in male mice. Furthermore, NO_2_ disrupted the process of glucose metabolism by decreasing the synthesis of hepatic glycogen and increasing the production of glucose, while also promoting lipid deposition through increased lipogenesis and uptake. As a result, it led to elevated levels of lipid oxidation and secretion [46].

Over the last three years, there has been widespread utilization of Mendelian randomization to establish a causal relationship between the two diseases. Recently, there has been a growing application of this method to investigate the link between pollutants and diseases. Liu C.X. et al. discovered casual links between PM_2.5_ and high blood pressure, T2D, and obesity [47]. Similarly, Li W.J. et al. identified positive associations between NO_x_ exposure and squamous cell lung cancer as well as esophageal cancer; between NO_2_ exposure and endometrial cancer as well as ovarian cancer; between PM_2.5_ exposure and ER+ breast cancer as well as ER- breast cancer; between PM_course_ exposure and glioma; and between PM_10_ exposure and mesothelioma as well as esophageal cancer [48]. Ning P.P. et al. found that PM_10_ was associated with an increased risk of Alzheimer’s disease [49]. Qiu S.Z. et al. found that, while the link between PM_2.5_ levels and lifespan was not statistically significant, PM_2.5_ exposure indirectly impacts lifespan through factors such as diastolic blood pressure (DBP), high blood pressure, angina pectoris, high cholesterol, and Alzheimer’s disease [50].

Our MR study has several advantages. Although there have been clinical cohort studies focused on air pollutants and NAFLD in various regions, as well as animal studies using different modeling methods, this is the first study to explore the influence of air pollutants on NAFLD using human GWAS data. Based on previously published authoritative epidemiological studies, the derivation set and validation sets were selected within the same race with different sample overlap rates, making the results comparable. Moreover, we used different MR methods and threshold conditions in the derivation set for discovery. At the same time, MVMR was used in the derivation set and UVMR was used in the validation sets for checking, which deepened the credibility of the results.

This study is subject to certain limitations. Recently, some researchers have questioned the use of MR to explore the relationship between air pollution and other diseases. The estimation of air pollutants was typically conducted by utilizing an individual’s residential address. Au Yeung believes that the use of MR is inappropriate because any genetic associations with air pollutants could easily be a reflection of hidden confounding, rendering the positive findings uninterpretable in relation to the research questions originally posed [51]. The application of MR in this article may also face such doubts, but it is still meaningful due to different study designs and assumption directions. Different from traditional MR, which attaches importance to evaluating the biological plausibility of genetics, MR in this article is only used as a mathematical statistical inference method, compared with traditional regression analysis methods used in epidemiology using the same exposure dataset. Presently, the prevailing techniques for sampling environmental air pollution rely on fixed-location and time-sampling methods, with the UKB serving as the most extensively utilized and expansive database in traditional epidemiological research. There may be varying degrees of confusion bias due to exposure and outcome datasets coming from the same source, so this article used two different outcome datasets as validation sets to reduce the interference of confounding factors. Meanwhile, when traditional epidemiology corrects confounding factors, the results may also be disrupted due to incomplete correction, so this article applied MVMR with confounding factor data from the same source as an exposure dataset for adjustment and obtained different results from the traditional epidemiology. Different statistical inference methods were used for the same dataset, resulting in different results and opposite conclusions, which made the finding valuable despite the use of MR potentially violating the assumption of biocompatibility to some extent. In addition, the MR analysis was conducted solely on individuals with European heritage, and this association might vary in individuals of different ancestries such as a South-East Asian population exposed to different levels of outdoor and indoor air pollution and with different distributions of confounders. Additionally, the data sets employed in our research had constraints regarding the air pollutant constituents. For instance, the absence of explicit PM_2.5_ component data in the summarized data hindered our ability to carry out additional subgroup analyses. Therefore, more research is still needed to determine whether different air pollutants can increase the risk of NAFLD and how.

## 5. Conclusions

In conclusion, this MR study provides genetic evidence for a null causal relationship between air pollution and NAFLD in the European population. Perhaps confounding elements have played an undeniable role in epidemiological studies that have found atmospheric pollutants were positively bound up with NAFLD. More human data and animal experiments are warranted to enhance our understanding in this area.

## Figures and Tables

**Table 1:** GWAS data sources of this MR study.

Exposure/Outcome	Dataset		Sample Size or Case/Control	NSNP	Unit	Population	Consortium/Cohort	Year
	GWAS ID	PMID						
Exposure								
PM_2.5_	ukb-b-10817	——	423,796	9,851,867	SD	European	MRC-IEU	2018
PM_2.5_ absorbance	ukb-b-11312	——	423,796	9,851,867	SD	European	MRC-IEU	2018
PM_2.5–10_	ukb-b-12963	——	423,796	9,851,867	SD	European	MRC-IEU	2018
PM_10_	ukb-b-18469	——	423,796	9,851,867	SD	European	MRC-IEU	2018
NO_2_	ukb-b-9942	——	456,380	9,851,867	SD	European	MRC-IEU	2018
NO_x_	ukb-b-12417	——	456,380	9,851,867	SD	European	MRC-IEU	2018
Alcohol intake frequency	ukb-b-5779	——	462,346	9,851,867	SD	European	MRC-IEU	2018
Outcome								
NAFLD	ebi-a-GCST90054782	34535985	4,761/373,227	9,097,254	Event	European	UK Biobank	2021
NAFLD	ebi-a-GCST90091033	34841290	8,434/770,180	6,784,388	Event	European	eMERGE, UK Biobank, FinnGen and Estonian Biobank	2021
NAFLD	finn-b-NAFLD	——	894/217,898	16,380,466	Event	European	FinnGen	2021

NSNP, number of Single Nucleotide Polymorphisms; PMID, PubMed ID; SD, standard deviation.

**Table 2:** MR analytical results of air pollution on NAFLD in the derivation dataset at level *p* < 1 × 10^−6^.

Exposure	Method	OR (95% CI)	*p*	NSNP	F Statistic Median (Min, Max)	*p* (Cochran’s Q Heterogeneity Test)	*p* (MR-Egger Intercept Test)	*p* (MR-PRESSO Global Test)
PM_2.5_	IVW	2.88 (0.73–11.33)	0.129	13	26.0 (24.1, 30.1)	0.027	0.504	0.030
	MR Egger	9.33 (0.25–347.19)	0.251					
	Weighted median	4.83 (1.03–22.65)	0.046					
	IVW-mre	2.88 (0.73–11.33)	0.129					
	MR-RAPS	3.11 (1.12–8.60)	0.029					
PM_2.5_ (Outlier-corrected)	IVW	4.26 (1.24–14.64)	0.021	12	25.9 (24.1, 30.1)	0.147	0.783	0.173
	MR Egger	6.53 (0.26–163.21)	0.280					
	Weighted median	5.59 (1.27–24.63)	0.023					
	IVW-mre	4.26 (1.24–14.64)	0.021					
	MR-RAPS	4.59 (1.56–13.44)	0.006					

Note: NSNP, number of Single Nucleotide Polymorphisms.

**Table 3:** MR analytical results of air pollution on NAFLD in the derivation dataset at level *p* < 1 × 10^−5^.

Exposure	Method	OR (95% CI)	*p*	NSNP	F Statistic Median (Min, Max)	*p* (Cochran’s Q Heterogeneity Test)	*p* (MR-Egger Intercept Test)	*p* (MR-PRESSO Global Test)
PM_2.5_	IVW	1.99 (1.22–3.22)	0.005	72	21.1 (19.6, 30.1)	0.321	0.317	0.313
	MR Egger	1.08 (0.30–3.88)	0.908					
	Weighted median	1.94 (0.98–3.80)	0.055					
	IVW-mre	1.99 (1.22–3.22)	0.005					
	MR-RAPS	2.06 (1.26–3.35)	0.004					
NO_2_	IVW	2.08 (1.27–3.40)	0.004	89	22.1 (19.5, 37.8)	0.028	0.099	0.031
	MR Egger	5.63 (1.58–20.07)	0.009					
	Weighted median	1.76 (0.92–3.36)	0.085					
	IVW-mre	2.08 (1.27–3.40)	0.004					
	MR-RAPS	2.17 (1.39–3.40)	0.001					

Note: NSNP, number of Single Nucleotide Polymorphisms.

## Data Availability

Only publicly available data were used in this study. Data sources and handling of these data are described in the Materials and Methods, Table 1, and Supplementary Tables S1–S4. The corresponding author can provide all the necessary data and materials supporting the findings of this work upon a reasonable request.

## Supplementary Materials

The following supporting information can be downloaded at: https://www.mdpi.com/article/10.3390/toxics12030228/s1, Table S1: Detailed information about exposure and outcome in the MR study; Table S2: Characteristics of the genetic instrument variables for the air pollution on NAFLD in the derivation dataset in the MR study at the genome-wide significance level (*p* < 1 × 10^−5^); Table S3: Characteristics of the SNPs correlated with the confounding factors for NAFLD or NAFLD directly at genome-wide significance level (*p* < 1 × 10^−5^); Table S4: MR analytical results of air pollution on NAFLD at different significance levels; STROBE-MR checklist.

## Abbreviations

PM	particulate matter
NO_2_	nitrogen dioxide
NO_x_	nitrogen oxides
NAFLD	non-alcoholic fatty liver disease
MAFLD	metabolic-associated fatty liver disease
MR	Mendelian randomization
UVMR	univariable Mendelian randomization
MVMR	multivariable Mendelian randomization
GWAS	Genome-Wide Association Study
SNPs	single nucleotide polymorphisms
IVs	instrumental variables
OR	odds ratio
CI	confidence interval
UKB	UK Biobank
MRC-IEU	UK Medical Research Council Integrative Epidemiology Unit
EGCUT	Estonian Genome Center of the University of Tartu
STROBE-MR	Strengthening observational studies using Mendelian randomization
MR-RAPS	MR-Robust Adjusted Profile Score
MR-PRESSO	MR-Pleiotropy RESidual Sum and Outlier
mre-IVW/IVW-mre	multiplicative random-effects inverse variance weighting
