# Supplementary material for: Investigation of the Association between Air Pollution and Non-Alcoholic Fatty Liver Disease in the European Population: A Mendelian Randomization Study
Source: Toxics. 2024 Mar 21;12(3):228. doi: 10.3390/toxics12030228 (PMC10974773; doi:10.3390/toxics12030228)
Supplement: Supplementary file 1 [file toxics-12-00228-s001.zip › Supplementary Material.pdf]

Supplementary Material

Investigation of the association between air pollution and non-alcoholic fatty liver disease in the European population: a Mendelian randomization study

Jing Yang[1†], Yaqi Zhang[1†], Yin Yuan[1,2†], Zhongyang Xie[1], Lanjuan Li[1*]

#These authors have contributed equally to this work and share first authorship
*** Correspondence: Corresponding Author: ljli@zju.edu.cn**

1 Supplementary Tables

Supplementary Table S1. Detailed information about exposure and outcome in the MR study.
Sheet1: Detailed information about exposures in UK Biobank.
Sheet2: Diagnostic codes used for identification of NAFLD in the MR studies.
Sheet3: Analytical methods used in datasets of NAFLD in the MR studies.
Notes: PM, Particulate matter; UKB, United Kingdom Biobank; Std.dev,standard deviation; ICD9 / ICD10: International Classification of Diseases Ninth and Tenth Revisions; READ V2 and READ V3, Clinical terminology system used in UK Primary Care settings (created by Dr James Read); †All READ V2 Codes also apply to READ V3 Codes; eMERGE Network:electronic Medical Records and Genomics (eMERGE) Network.

Supplementary Table S2. Characteristics of the genetic instrument variables for the air pollution on non-alcoholic fatty liver disease in the derivation dataset in the Mendelian randomization study at the genome-wide significance level ($p < 1 \times 10^{-5}$).
Sheet1: Characteristics of the genetic instrument variables for $PM_{2.5}$ on non-alcoholic fatty liver disease in the derivation dataset in the Mendelian randomization study at level $p < 1 \times 10^{-5}$.
Sheet2: Characteristics of the genetic instrument variables for $PM_{2.5}$ absorbance on non-alcoholic fatty liver disease in the derivation dataset in the Mendelian randomization study at level $p < 1 \times 10^{-5}$.
Sheet3: Characteristics of the genetic instrument variables for $PM_{2.5\text{-}10}$ on non-alcoholic fatty liver disease in the derivation dataset in the Mendelian randomization study at level $p < 1 \times 10^{-5}$.
Sheet4: Characteristics of the genetic instrument variables for PM_{10} on non-alcoholic fatty liver disease in the derivation dataset in the Mendelian randomization study at level $p < 1 \times 10^{-5}$.
Sheet5: Characteristics of the genetic instrument variables for nitrogen dioxide on non-

alcoholic fatty liver disease in the derivation dataset in the Mendelian randomization study at level $p < 1 \times 10^{-5}$.

Sheet6: Characteristics of the genetic instrument variables for nitrogen oxides on non-alcoholic fatty liver disease in the derivation dataset in the Mendelian randomization study at level $p < 1 \times 10^{-5}$.

Supplementary Table S3. Characteristics of the SNPs correlated with the confounding factors for NAFLD or NAFLD directly at genome-wide significance level ($p < 1 \times 10^{-5}$) .

Sheet1: Characteristics of the SNPs as the genetic instrument variables for $PM_{2.5}$ correlated with the confounding factors for NAFLD or NAFLD directly at level $P < 1 \times 10^{-5}$.

Sheet2: Characteristics of the SNPs as the genetic instrument variables for $PM_{2.5}$ absorbance correlated with the confounding factors for NAFLD or NAFLD directly at level $p < 1 \times 10^{-5}$.

Sheet3: Characteristics of the SNPs as the genetic instrument variables for $PM_{2.5\text{-}10}$ correlated with the confounding factors for NAFLD or NAFLD directly at level $p < 1 \times 10^{-5}$.

Sheet4: Characteristics of the SNPs as the genetic instrument variables for PM_{10} correlated with the confounding factors for NAFLD or NAFLD directly at level $p < 1 \times 10^{-5}$.

Sheet5: Characteristics of the SNPs as the genetic instrument variables for nitrogen dioxide correlated with the confounding factors for NAFLD or NAFLD directly at level $p < 1 \times 10^{-5}$.

Sheet6: Characteristics of the SNPs as the genetic instrument variables for nitrogen oxides correlated with the confounding factors for NAFLD or NAFLD directly at level $p < 1 \times 10^{-5}$.

Supplementary Table S4. MR analytical results of air pollution on non-alcoholic fatty liver disease at different significance levels.

Sheet1: MR original analytical results of air pollution on non-alcoholic fatty liver disease in the derivation dataset at level $p < 5 \times 10^{-8}$.

Sheet2: MR analytical results of air pollution on non-alcoholic fatty liver disease in the derivation dataset at level $p < 5 \times 10^{-8}$ without SNPs in Table S2.

Sheet3: MR original analytical results of air pollution on non-alcoholic fatty liver disease in the derivation dataset at level $p < 1 \times 10^{-6}$.

Sheet4: MR analytical results of air pollution on non-alcoholic fatty liver disease in the derivation dataset at level $p < 1 \times 10^{-6}$ without SNPs in Table S2.

Sheet5: MR original analytical results of air pollution on non-alcoholic fatty liver disease in the derivation dataset at level $p < 1 \times 10^{-5}$.

Sheet6: MR analytical results of air pollution on non-alcoholic fatty liver disease in the derivation dataset at level $p < 1 \times 10^{-5}$ without SNPs in Table S2.

Sheet7: Bias, Type 1 error rate for Mendelian randomization with sample overlap and power in the derivation dataset.

Sheet8: MR analytical results of air pollution on non-alcoholic fatty liver disease in the validation datasets at level $p < 1 \times 10^{-5}$ without SNPs in Table S2.

Notes:

IVW-mre:Inverse variance weighted-multiplicative random effects; MR-RAPS:Mendelian Randomization-Robust Adjusted Profile Score; MR-PRESSO:Mendelian Randomization-Pleiotropy RESidual Sum and Outlier; OR:odds ratio; OR_low: The lower of 95% confidence interval of OR; OR_up: The upper of 95% confidence interval of OR; NA:Not available.

2 STROBE-MR checklist

Strengthening observational studies using Mendelian randomization (STROBE-MR) checklist.
